# Substance use and nicotine dependence in persistent, remittent, and late-onset ADHD: a 10-year longitudinal study from childhood to young adulthood

**DOI:** 10.1186/s11689-018-9260-y

**Published:** 2018-12-27

**Authors:** Shahrzad Ilbegi, Annabeth P. Groenman, Arnt Schellekens, Catharina A. Hartman, Pieter J. Hoekstra, Barbara Franke, Stephen V. Faraone, Nanda N. J. Rommelse, Jan K. Buitelaar

**Affiliations:** 1 Department of Cognitive Neuroscience, Donders Institute for Brain, Cognition and Behaviour, Radboud University Medical Center, Nijmegen, The Netherlands; 2 Department of Child and Adolescent Psychiatry, University of Groningen, University Medical Center Groningen, Groningen, The Netherlands; 3 Department of Psychiatry, Donders Institute for Brain, Cognition and Behaviour, Radboud University Medical Center, Nijmegen, The Netherlands; 4 Nijmegen Institute for Scientist Practitioners in Addiction, Nijmegen, The Netherlands; 5 Department of Psychiatry, University of Groningen, University Medical Center Groningen, Groningen, The Netherlands; 6 Department of Human Genetics, Donders Institute for Brain, Cognition and Behaviour, Radboud University Medical Center, Nijmegen, The Netherlands; 7 Departments of Psychiatry and of Neuroscience and Physiology, SUNY Upstate Medical University, Syracuse, USA; 8 Karakter Child and Adolescent Psychiatry University Center, Nijmegen, The Netherlands; 9 Donders Center for Neuroscience, Radboud University Medical Center, PO Box 9101, 6500 HB Nijmegen, The Netherlands

**Keywords:** ADHD course, Late-onset ADHD, Substance use disorders, Nicotine dependence

## Abstract

**Background:**

Attention-deficit/hyperactivity disorder (ADHD) is associated with substance use disorders (SUD; alcohol and/or drug dependence) and nicotine dependence. This study aims to advance our knowledge about the association between SUD, nicotine dependence, and the course of ADHD (persistent versus remittent ADHD and late-onset ADHD).

**Methods:**

ADHD, SUD, and nicotine dependence were longitudinally assessed (mean age at study entry 11.3 years, mean age at follow-up 21.1 years) using structured psychiatric interviews and multi-informant questionnaires in a subsample of the Dutch part of the International Multicenter ADHD Genetics study. Individuals with persistent ADHD (*n* = 62), remittent ADHD (*n* = 12), late-onset ADHD (*n* = 18; age of onset after 12 years), unaffected siblings (*n* = 50), and healthy controls (*n* = 47) were assessed. Hazard ratios (HR) with 95% confidence intervals (CIs) were estimated by Cox regression and adjusted for clustered family data, gender, follow-up length, and current age.

**Results:**

Individuals with persistent ADHD were at significantly higher risk of development of SUD relative to healthy controls (HR = 4.56, CI 1.17–17.81). In contrast, levels of SUD in those with remittent ADHD were not different from healthy controls (HR = 1.00, CI .07–13.02). ADHD persisters had also higher prevalence rates of nicotine dependence (24.2%) than ADHD remitters (16.7%) and healthy controls (4.3%). A similar pattern was found in initially unaffected siblings who met ADHD criteria at follow-up (“late-onset” ADHD); they had also a higher prevalence of SUD (33%) compared to stable unaffected siblings (20%) and were at significantly increased risk of development of nicotine dependence compared to healthy controls (HR = 13.04, CI 2.08–81.83).

**Conclusions:**

SUD and nicotine dependence are associated with a negative ADHD outcome. Results further emphasize the need for clinicians to comprehensively assess substance use when diagnosing ADHD in adolescents and adults.

**Electronic supplementary material:**

The online version of this article (10.1186/s11689-018-9260-y) contains supplementary material, which is available to authorized users.

## Background

Attention-deficit/hyperactivity disorder (ADHD) is a neurodevelopmental disorder with onset in childhood; the disorder is characterized by a heterogeneous etiology and developmental course. Although severity of symptoms, especially of hyperactivity-impulsivity, often diminishes over time, prospective longitudinal studies have shown that the impairing symptoms of the disorder persist into adulthood in approximately two thirds of children with ADHD, with associated impairment across multiple domains [1]. Recent studies have challenged the established notion of ADHD as an exclusive childhood-onset disorder (onset prior to 12 years of age) and reported onset of ADHD in adolescence [2] and adulthood [3–5].

Various risk factors have been associated with a more persistent course, such as higher ADHD symptom severity, presence of comorbidity (in particular conduct and mood disorders), family history of ADHD, and psychosocial and environmental adversities [6]. Longitudinal studies of children with ADHD have consistently identified early substance use in adolescence as an important negative outcome of the disorder [7–9]. Although many studies have examined the link between ADHD and the onset of substance use disorders (SUD; alcohol and/or drug use disorder) and nicotine dependence (ND) [10, 11], few have investigated the association between SUD/ND and the course of ADHD over time. While comorbid conduct disorder and oppositional defiant disorder among children with ADHD increase the risk for SUD [12], several studies have shown that ADHD is an independent risk factor for the development of SUD in adolescence and adulthood [10, 11]. Moreover, ND may increase the risk of other SUD [13]. For example, in a longitudinal study, Biederman et al. [14] found that ADHD youth who smoked cigarettes were more likely to subsequently use other substances and to develop SUD, compared to other ADHD youth. This risk is further increased when ADHD symptoms persist into adulthood, with higher prevalence rates of SUD found among individuals with persistent than among those with remitted ADHD [10, 15].

It is unclear whether there is also a link between late-onset ADHD, referring to the onset of ADHD after 12 years of age, and SUD/ND. Several birth cohort studies have reported a 2.5–10.7% prevalence of a form of ADHD that first emerged in mid/late adolescence or (young) adulthood, so-called late-onset ADHD [3–5]. ADHD with onset after age 12 had patterns of psychiatric comorbidity, functional impairment, familial transmission, and intelligence similar to childhood-onset ADHD [2]. Careful assessment of ADHD symptoms that emerged after childhood is crucial, since false positive cases of adult-onset ADHD are common [16]. Moreover, the reliability of an age at onset assessment could be increased by using multiple informants or by selecting (young) adult participants from prospective follow-up studies.

The present study examined whether the course of ADHD is associated with increased risks for developing SUD (alcohol and/or drug use disorder) and ND from childhood through young adulthood. We report findings from a 10-year prospective, longitudinal study of a subsample of the Dutch International Multicenter ADHD Genetics (IMAGE) study cohort, including individuals with persistent ADHD (*n* = 62), remittent ADHD (*n* = 12), late-onset ADHD (*n* = 18), unaffected siblings (*n* = 50), and healthy controls (*n* = 47). Given the extant literature that ADHD is a risk factor for SUD, we hypothesized that the persistent ADHD group would show higher rates of SUD and ND compared to the remittent ADHD group, while we expected higher prevalence rates of SUD and ND in the late-onset ADHD group compared to the stable unaffected groups (unaffected siblings and healthy controls). By including a high-risk group of full biological siblings of ADHD probands, this study allowed us to investigate the ADHD course and development of SUD and ND over time in this group, characterized by increased genetic and/or environmental risk for ADHD.

## Methods

### Participants

All participants (*n* = 189) were a subsample of the Dutch node of the IMAGE study, with initial assessment taking place between 2003 and 2006 (wave 1) in VU University Amsterdam and Radboud University Medical Center Nijmegen. The IMAGE study recruited families with at least one child with clinically diagnosed ADHD combined type and their siblings regardless of ADHD diagnosis. Family members were of European Caucasian descent. Inclusion criteria applying to both probands and siblings included IQ≥ 70, no diagnosis of autism, epilepsy, general learning difficulties, brain disorders, and known genetic disorders. Additional healthy control participants were recruited from primary and high schools from the same geographical regions as the participating ADHD probands and siblings. To assess SUD, all families were re-invited for an intermediate follow-up study (wave 2) and follow-up assessment 2 (wave 3) and 5 years later (wave 4; only the Nijmegen sub-cohort). The present study included all children and adolescents who participated in wave 1, 2, and 4 (in total *N* = 189). For the current analysis, wave 3 was left out considering no information about SUD/ND was collected. Note that affected siblings were grouped in the ADHD affected group. Results for ADHD vs. unaffected siblings vs. healthy controls based on the diagnosis at baseline are presented in Additional file 1: Tables S1, S2, and Figure S1.

### Measures

#### Diagnostic assessment

To determine ADHD diagnosis at wave 1 and wave 4, all participants in the study were assessed similarly, using the standard procedures of the IMAGE project, described fully elsewhere [17]. The long version of Conners’ Parent (CPRS-R:L) and Teacher Rating Scales (CTRS-R:L; [18]) were used to identify and quantify ADHD symptoms; *T*-scores ≥ 63 on the Conners’ ADHD subscales inattention (L), hyperactivity/impulsivity (M), and total symptoms (N) and scores ≥ 90th percentile on the Strengths and Difficulties Questionnaire (SDQ; [19]). Hyperactivity subscale were considered clinical. At wave 4, the Adult ADHD Rating Scales-Self-Report: Long Version (CAARS-S:L) was added for participants ≥ 18 years. Participants scoring clinically on any of these subscales were administered a diagnostic interview. The diagnostic interview changed from the Parental Account of Children’s Symptoms (PACS; [20]) at wave 1 to the Schedule for Affective Disorders and Schizophrenia for School-Age Children (K-SADS; [21]) at wave 4, both semi-structured, standardized, investigator-based interviews with the parents as informants. When children were 12 years or older, the K-SADS was also administered individually (wave 4). Participants with elevated scores on any of the screen items were administered the full ADHD interview. A diagnostic algorithm was used to establish ADHD status as defined by Diagnostic and Statistical Manual of Mental Disorders: (4th ed.; DSM-IV; [22]) and DSM-5 (5th ed.; [23]). ADHD types (combined, predominantly inattentive, or predominantly hyperactive/impulsive type) were established following DSM-IV (wave 1) and DSM-5 (wave 4) criteria. Comorbidities were assessed using the PACS at baseline and using the K-SADS at follow-up. Classifications in both interviews were established according to DSM-IV (wave 1) or DSM-5 (wave 4) criteria for comorbid conduct disorder and oppositional defiant disorder. Classifications of DSM-IV anxiety, mood, and tic disorders were established in the K-SADS at follow-up. Persistence of ADHD was defined as meeting full DSM-IV criteria of ADHD/C at baseline and meeting full DSM-5 criteria of ADHD regardless of presentation at follow-up. Remission of ADHD was defined as meeting full criteria of ADHD/C at baseline and not meeting criteria of ADHD, any type, at follow-up. Late-onset ADHD was defined as meeting full DSM-5 criteria for ADHD at follow-up, with onset after 12 but prior to 18 years of age, and not meeting criteria of ADHD, any type, at baseline. Unaffected siblings and healthy controls were defined as not meeting criteria of (subthreshold) ADHD, any type, at baseline and follow-up. Subthreshold ADHD cases were excluded from analyses. For both the PACS and K-SADS, interviewers underwent comprehensive training by a team under the supervision of E. Taylor at the London Institute of Psychiatry (PACS) or J. Buitelaar at the Donders Institute for Brain, Cognition and Behaviour, Radboud University Medical Center, Nijmegen (K-SADS). The interviewers were trained child psychiatrists, child psychologists, or clinically trained researchers.

#### Substance use disorder assessment

For SUD assessment, a number of questionnaires were completed by participants at wave 2 and wave 4. The Drug Abuse Screening Test-20 [24] was used to assess drug use disorders. Scores on this questionnaire may range from 0 to 20. A cutoff of 5 was used to identify possible drug use disorders [24]. The Fagerström Test for Nicotine Dependence [25] was used to assess ND. Scores on this questionnaire may vary between 0 and 10. A cutoff of 2 was used to identify ND. Age of first nicotine use was also assessed in this questionnaire. The Short Michigan Alcohol Screening Test [26] was administered to identify possible alcohol use disorders. Scores varied between 0 and 13, and a cutoff of 4 was used to define alcohol dependence. At wave 2, participants were provided with a personal return envelope to increase trust and to ensure confidentiality of sensitive information. A best-estimate diagnosis of SUD was considered present if either alcohol or drug use disorder according to the DSM-IV criteria was present.

#### Anxiety and emotional lability

*T*-scores on the CPRS-R:L subscales anxious/shy (D) and emotional lability (J) were used to assess levels of anxiety and emotional lability.

### Procedure

At baseline, families were recruited from clinics and via advertisements. Testing took place at the Donders Institute and Radboudumc in Nijmegen. All ratings of behavioral functioning pertained the participant’s functioning off medication. Families were financially compensated for participating in the study.

### Statistical analysis

All analyses were conducted using SPSS version 24.0 (IBM SPSS Statistics for Macintosh, Version 24.0). Participants were divided into five groups based on prospective reports of ADHD symptoms (persistent, remittent and late-onset ADHD, (stable) unaffected siblings, and healthy controls). Analyses of variance were performed to assess whether groups (persistent vs. remittent and late-onset ADHD vs. unaffected siblings) differed on IQ at baseline, age at follow-up, follow-up interval, emotional lability, and anxiety *T*-scores of the CPRS-R:L and *T*-scores on the ADHD scales of the CPRS-R:L. A chi-square test assessed whether groups differed in the proportion of males. Any variables showing significant differences between groups were included as covariates. Group differences in risk of developing SUDs and ND were assessed using Cox proportional hazard models. Correction for clustered (family) data was performed using robust standard errors [27]. The model used the age of first substance or nicotine use as the survival time for the cases (i.e., those with either SUDs or ND) and current age was used as the time of censoring for the non-cases. The event is defined as lifetime SUD or ND. The effect of stimulant medication use (yes/no) on the development of SUDs or ND was assessed by using this variable as a group in Cox regression using SUDs or ND as outcome variables. Finally, we checked whether the associations between ND and SUD and course of ADHD were not driven by group differences in levels of anxiety and emotional lability.

## Results

### Attrition analyses

Selective attrition was investigated by comparing participants successfully followed up (*n* = 189) with participants lost to follow up on variables reported in this study available at baseline. No significant group differences were found (.14 < *p* < .99).

### Demographic and clinical characteristics

Additional file 1: Table S3 describes the demographic and clinical characteristics of the five groups. Of 74 children with ADHD combined type at baseline, 62/74 (84%) persisted in a full ADHD diagnosis (50% ADHD inattentive-type, 9.7% ADHD hyperactive/impulsive-type, 40.3% ADHD combined type) and 12/74 (16%) remitted from the disorder at follow-up. Of note, 18/68 (26%) of the unaffected siblings at baseline met diagnostic criteria of ADHD at follow-up (14 adolescent-onset and 4 adult-onset), while no late-onset (adolescent nor adult-onset) ADHD was found in healthy controls (*n* = 47). There were small but statistically significant group differences in current age, follow-up interval, gender, and IQ. All subsequent analyses were statistically corrected for current age, follow-up interval, and gender.

No significant differences in anxiety and emotional lability scores were found between siblings with late-onset ADHD and (stable) unaffected siblings at baseline (see Additional file 1: Table S1 for details). Between baseline and follow-up, individuals with persistent and remittent ADHD, (stable) unaffected siblings, and healthy controls showed a decrease in anxiety and emotional lability symptom severity scores, while the late-onset ADHD group remained stable in their levels of anxiety and emotional lability. Levels of emotional lability in the late-onset ADHD group were higher than in (stable) unaffected siblings and similar to the persistent ADHD group at follow-up, but not in the clinical range. It is therefore unlikely that this explains clinical levels of ADHD symptoms in young adulthood (see Additional file 1: Table S3 for details).

### Risk for substance use disorder and nicotine dependence in persisters and remitters

**Fig. 1:**
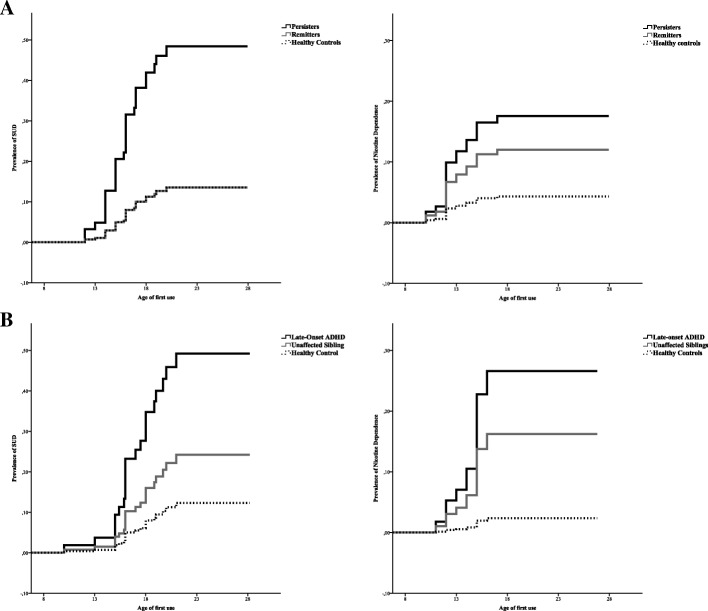
Cumulative lifetime risk for any substance use disorder and nicotine dependence in **a** persisters, remitters and healthy controls and **b** late-onset ADHD, unaffected siblings and healthy controls

A main group effect was found when comparing persisters with remitters and healthy controls (Wald *F* = 4.25, *p* = .018). Individuals with persistent ADHD were 4.6 times (95% CI 1.17–17.81) more likely to develop a SUD compared to healthy controls, whereas remitters did not differ in risk compared to healthy controls (HR = 1.0, 95% CI .07–13.02). There was no significant difference in risk of developing a SUD between persisters and remitters, although the small sample size and wide confidence interval likely contributed to this (HR = .22, 95% CI .03–13.02). No main effect of group was found when persisters, remitters, and healthy controls were compared on their risk of developing nicotine dependence (Wald *F* = 1.48, *p* = .23), although, again, the overall pattern of findings suggests that persisters and remitters had higher nicotine dependence than healthy controls; see Table 1 and Fig. 1a.

**Table 1:** Prevalence rates of SUD and ND in ADHD persisters, remitters, and healthy controls

	Prevalence rates						Hazard ratios					
	Persisters *n* = 62		Remitters *n* = 12		HC *n* = 47		Persisters vs. HC		Persisters vs. Remitters		Remitters vs. HC	
	*n*	%	*n*	%	*n*	%	HR	95% CI	HR	95% CI	HR	95% CI
Substance use disorder	22	35.5	1	8.3	6	12.8	4.56*	1.17–17.81	.22	.03–1.57	1.00	.07–13.02
Nicotine dependence	15	24.2	2	16.7	2	4.3	4.38	.77–25.02	.66	.13–3.41	2.90	.29–29.57

*SUD* substance use disorder, *ND* nicotine dependence, *ADHD* attention deficit hyperactivity disorder, *HC* healthy controls. Hazard ratios (*HR*) were calculated using Cox proportional hazard regression. All comparisons were corrected for gender and follow-up interval in years. *95% CI* 95% confidence interval. *Significant at *p* < 0.05

### Risk for substance use disorder and nicotine dependence in late-onset ADHD and stable unaffected siblings

No significant difference between late-onset ADHD, unaffected siblings, and healthy controls was found when comparing them on the risk of developing a SUD (Wald *F* = 2.28, *p* = .11). Again, the pattern of findings suggests that late-onset ADHD had higher SUD levels (see Fig. 1). However, a main effect of group was found when looking at nicotine dependence (Wald *F* = 4.40, *p* = .016). Those with late-onset ADHD were at increased risk of developing nicotine dependence compared to healthy controls (HR = 13.04, 95% CI 2.08–81.83), but not compared to unaffected siblings (HR = .57, 95% CI .21–1.6). Unaffected siblings and healthy controls did not significantly differ in their risk of developing nicotine dependence, although the small sample size and wide confidence interval likely contributed to this, with the pattern of findings suggesting increased prevalence of nicotine dependence in unaffected siblings compared to healthy controls (HR = 7.47, 95% CI .91–61.13; also see Table 2 and Fig. 1b).

**Table 2:** Prevalence rates of SUD and ND in late-onset ADHD, (stable) unaffected siblings, and healthy controls

	Prevalence rates						Hazard ratios					
	LO-ADHD *n =* 18		US *n* = 50		HC *n* = 47		LO-ADHD vs. HC		LO-ADHD vs. US		US vs. HC	
	*n*	%	*n*	%	*n*	%	HR	95% CI	HR	95% CI	HR	95% CI
Substance use disorder	6	33.3	10	20.0	6	12.8	5.15	1.04–25.66	.41	.14–1.23	2.11	.51–8.73
Nicotine dependence	5	27.8	8	16.0	2	4.3	13.04*	2.08–81.83	.57	.21–1.60	7.47	.91–61.13

*SUD* substance use disorder, *ND* nicotine dependence, *LO-ADHD* late-onset attention deficit hyperactivity disorder, *US* unaffected siblings, *HC* healthy controls. Hazard ratios (*HR*) were calculated using Cox proportional hazard regression. All comparisons were corrected for gender and follow-up interval in years. 95% *CI* 95% confidence interval. *Significant at *p* < 0.05

### Medication use

No significant effect of stimulant medication use was found on the development of SUDs (Wald *F* = .72, *p* = .40) or nicotine dependence (Wald *F* = .47 *p* = .49).

## Discussion

To our knowledge, this study provides the first data on the association between SUD/ND and the course of ADHD among probands with ADHD and their biological siblings in a 10-year prospective longitudinal study. Results showed that ADHD persisters were at significantly higher risk for the subsequent development of SUD relative to healthy controls, in contrast to a similar level of SUD in ADHD remitters vs healthy controls. ADHD persisters had also higher (albeit non-significant) prevalence rates of ND than ADHD remitters and healthy controls. A similar pattern of results was found for those with late-onset ADHD who had a higher prevalence of SUD compared to healthy controls, while no higher prevalence rates of SUD were found in stable unaffected siblings compared to healthy controls. Further, those with late-onset ADHD were at significantly higher risk for the subsequent development of ND compared to healthy controls, in contrast to a similar level of SUD in stable unaffected siblings versus healthy controls*.* Although not all results reached statistical significance, the pattern of findings suggests SUD and ND are associated with a negative ADHD outcome (i.e., persistent ADHD and/or late-onset ADHD) and further emphasize the need for clinicians to make a comprehensive assessment of substance use when diagnosing ADHD.

Previous research has suggested that the persistence of ADHD is a risk factor for the development of SUD [15]. Our study replicated that finding and extended the analysis by showing the same pattern of results in biological siblings with late-onset ADHD. The findings suggest that late-onset ADHD is also associated with ND. ND is often described as a gateway drug to illicit drug use [28], and studies consistently identify the increased risk of ND in individuals with ADHD [13]. In our samples, both persistent and remitted ADHD had higher rates of ND than the controls, although the effects were not significant. This is likely due to small sample sizes and hence limited statistical power. Given that SUD and ND might be associated with adverse outcome of ADHD, identification of early risk factors and preventive interventions in children at risk for a persistent course of ADHD as well as their biological siblings might be crucial.

Disentangling the nature of the association between ADHD and SUD/ND remains challenging. Although the literature is consistent in showing that the onset of ADHD precedes the development of SUD, high levels of substance use in adolescence may adversely affect still-maturing prefrontal brain regions leading to behavior regulation deficits associated with ADHD [29]. In this perspective, substance use could negatively influence the course of ADHD in addition to the widely accepted ADHD-to-substance use pathway. Alternatively, because SUD often manifests in adolescence and young adulthood, the SUDs itself might elicit symptoms of ADHD and be mistakenly identified as “late-onset of ADHD” [30]. A third possible explanation for the association between ADHD course and SUD/ND is that the relation between ADHD and SUD/ND is largely explained by a common third factor that increases both the risk for a detrimental ADHD course as well as increase the risk for SUD/ND, for instance, environmental factors and/or shared genetic lability to both disorders [31, 32].

Our findings offer some support for the possibility of late-onset ADHD, a proportion of biological siblings unaffected at baseline met DSM-5 criteria of ADHD in young adulthood, with age of onset after 12 but prior to 18 years of age. Since all participants were comprehensively assessed on ADHD and comorbid disorders by multi-informant questionnaires and interviews in childhood, as well as in (young) adulthood, it is unlikely that these cases represented individuals with undetected childhood symptoms (i.e., late-identified rather than late-onset) [33–35] or false positive cases of late-onset ADHD. Although emotional lability scores at follow-up were higher in the late-onset ADHD group compared to the stable unaffected siblings group, these levels were not in the clinical range. An explanation for late-onset ADHD in biological siblings might be that unaffected siblings carry vulnerable heritable traits for ADHD and often already have increased levels of ADHD symptoms in childhood. However, their ADHD symptoms might not be severe enough to merit a clinical diagnosis. Clinical levels of ADHD might manifest later in life, when the demands of life increase or until they can no longer rely on or compensate by protective factors, such as high cognitive ability [4]. Future studies should investigate mechanisms associated with later onset ADHD in adolescence or adulthood, e.g., neuropsychological profile that possibly could differentiate individuals developing late-onset ADHD versus stable unaffected individuals.

Previous family studies of ADHD have shown that ADHD and SUD co-aggregate in families [30, 36]. In accordance with these findings, we found an increased risk of SUD among unaffected siblings of ADHD probands. Importantly, these findings indicate that there is no direct relationship between ADHD and SUD and that shared genetic lability and/or family environment risk factors to both disorders might contribute to the development of both. Previous findings from our study showed no increased risk of SUD in unaffected biological siblings of ADHD probands [11]; however, our participants in this study were still relatively young (mean 17 years) and might not have completely traversed the developmental pathway to substance use and ND. The present finding supports the notion that SUD increases during the late adolescent and young adult years. Taken together, our findings support the hypothesis that ADHD is a familial risk factor for SUD and that biological siblings represent a group who are at high risk for the subsequent development of both SUD and late-onset ADHD.

Some methodological limitations should be taken into account in the interpretation of the results. Given that several of the subgroup samples were relatively small, detecting significant group differences was more difficult. However, our results were in the expected direction, indicating that significant results are expected with a larger sample. Furthermore, SUD and ND were assessed by self-reports. We used adult cutoff scores for the self-report questionnaires, while not all subjects reached adulthood at the time of assessment. Although this approach would not have biased results to finding spurious case-control differences, it may have influenced our estimates of prevalence.

## Conclusions

This study contributes to the understanding of the association between substance use and the course of ADHD over time. In particular, children with persistent ADHD have higher risks of developing alcohol and/or drug dependence over time compared to healthy controls, while this was not found for individuals with remittent ADHD. The same pattern of findings was found for siblings developing late-onset ADHD versus those who remained unaffected. This suggests that SUD and ND seem to be associated with a negative ADHD outcome. Although the mechanisms governing these associations are not yet fully understood, the findings from this study underscore the clinical and public health significance of SUD in the course of ADHD. This study emphasizes the importance of preventive interventions in biological siblings, considering their increased risks of developing both SUD and ADHD.

## Additional file

Additional file 1: **Table S1.** Demographic and clinical characteristics of children with attention-deficit/hyperactivity disorder (ADHD), their unaffected siblings (US) and healthy controls (HC). **Table S2.** Prevalence rates of substance use disorder and nicotine dependence in children with attention deficit hyperactivity disorder (ADHD), their unaffected siblings (US) and healthy controls (HC). **Figure S1.** Cumulative life-time risk for any substance use disorder (SUD) and nicotine dependence in Attention-deficit hyperactivity disorder (ADHD) probands (*n* = 74), their unaffected siblings (*n* = 68) and healthy controls (*n* = 47) at baseline. All comparisons were corrected for gender and follow-up interval. **Table S3.** Demographic and clinical characteristics of the Attention-deficit/hyperactivity disorder (ADHD) persistent, remittent, late-onset (LO), unaffected siblings (US) and healthy controls (HC) group. (DOCX 137 kb)

## Abbreviations

ADHD: Attention-deficit/hyperactivity disorder
CAARS-S:L: Conners’ Adult ADHD Rating Scales-Self-Report: Long Version
CI: Confidence interval
CPRS-R:L: Conners’ Parent Rating Scale: Long Version
CTRS-R:L: Conners’ Teacher Rating Scale: Long Version
DSM-5: Diagnostic and Statistical Manual of Mental Disorders 5th edition
DSM-IV: Diagnostic and Statistical Manual of Mental Disorders 4th edition
HR: Hazard ratios
IMAGE: International Multicenter ADHD Genetics
IQ: Intelligent Quotient
K-SADS: Schedule for Affective Disorders and Schizophrenia for School-Age Children
ND: Nicotine dependence
PACS: Parental Account of Children’s Symptoms
SPSS: Statistical Package for the Social Sciences
SUD: Substance use disorders
