# Supplementary material for: Substance use and nicotine dependence in persistent, remittent, and late-onset ADHD: a 10-year longitudinal study from childhood to young adulthood
Source: J Neurodev Disord. 2018 Dec 27;10:42. doi: 10.1186/s11689-018-9260-y (PMC6307241; doi:10.1186/s11689-018-9260-y)
Supplement: Supplementary file 1 — Table S1. Demographic and clinical characteristics of children with attention-deficit/hyperactivity disorder (ADHD), their unaffected siblings (US) and healthy controls (HC). Table S2. Prevalence rates of substance use disorder and nicotine dependence in children with attention deficit hyperactivity disorder (ADHD), their unaffected siblings (US) and healthy controls (HC). Figure S1. Cumulative life-time risk for any substance use disorder (SUD) and nicotine dependence in Attention-deficit hyperactivity disorder (ADHD) probands (n = 74), their unaffected siblings (n = 68) and healthy controls (n = 47) at baseline. All comparisons were corrected for gender and follow-up interval. Table S3. Demographic and clinical characteristics of the Attention-deficit/hyperactivity disorder (ADHD) persistent, remittent, late-onset (LO), unaffected siblings (US) and healthy controls (HC) group. (DOCX 137 kb) [file 11689_2018_9260_MOESM1_ESM.docx]

**Supplements**

*Risk for substance use disorder and nicotine dependence in ADHD cases and their unaffected siblings*

When comparing ADHD probands (*n* = 74), their biological siblings (*n* = 68) and healthy controls (*n* = 47; for group characteristic see Table S1) on their risk of developing SUDs a main effect of group was found (Wald *F* = 3.31, *p* = .041). Individuals with ADHD were 4.6 times [95% CI: 1.43-14.9] more likely to develop a SUD compared to healthy controls, whereas ADHD unaffected siblings were 3.2 times 95% (CI: 1.07-9.78) more likely to develop a SUD compared to healthy controls. There were no significant differences in the risk of developing a SUD between probands and their siblings (HR= .7, 95% CI: .35-1.38). No main effect of group was found (Wald *F* = 2.35, *p* = .102) when comparing ADHD probands, their siblings and healthy controls on their risk of developing nicotine dependence (also see Table S2 and Figure S1).

| *Table S1.*  Demographic and clinical characteristics of children with attention-deficit/hyperactivity disorder (ADHD), their unaffected siblings (US) and healthy controls (HC). | | | | | | | | |
| --- | --- | --- | --- | --- | --- | --- | --- | --- |
|  | ADHD *n* = 74 | | US *n* = 68 | | HC *n* = 47 | |  |  |
|  | *M* | SD | *M* | SD | *M* | SD | Test Statistic | Post-hoc |
| *Wave 1* |  |  |  |  |  |  |  |  |
| *Demographic* |  |  |  |  |  |  |  |  |
| Age | 11.26 | 2.82 | 11.15 | 3.71 | 11.5 | 3.29 | *p* = 0.859 | 1=2=3 |
| Gender, *N* male (%) | 56 | 75.7 | 32 | 47.1 | 21 | 44.7 | χ2 = 16.21,  *p* < 0.001 | 1>2=3 |
| Estimated full-scale IQ | 98 | 12 | 101 | 12 | 110 | 10 | *p* < 0.001 | 1=2<3 |
| *ADHD severity* |  |  |  |  |  |  |  |  |
| CPRS-R:L inattentive symptom severity T-score | 71.3 | 9.09 | 50.81 | 9.09 | 45.22 | 3.91 | *p* < 0.001 | 1>2>3 |
| CPRS-R:L hyperactive/impulse symptom severity T-score | 77.68 | 10.61 | 53.47 | 11.88 | 45.78 | 3.37 | *p* < 0.001 | 1>2>3 |
| CPRS-R:L total symptom severity T-score | 76.27 | 9.02 | 52.22 | 10.81 | 44.89 | 3.16 | *p* < 0.001 | 1>2>3 |
| Inattentive Symptom Count | 7.83 | .95 | - | - | - | - | - | - |
| Hyperactive/Impulsive Symptom Count | 8 | 1.46 | - | - | - | - | - | - |
| Wave 4 |  | |  | |  | |  |  |
| *Demographic* |  |  |  |  |  |  |  |  |
| Age | 21.08 | 2.97 | 20.88 | 3.55 | 19.42 | 3.33 | *p* = 0.018 | 1=2>3 |
| Estimated full-scale IQ | 98 | 19 | 105 | 16 | 114 | 13 | *p* < 0.001 | 1<2<3 |
| *ADHD severity* |  |  |  |  |  |  |  |  |
| CPRS-R:L inattentive symptom severity T-score | 60.83 | 12.46 | 50 | 10.74 | 44.2 | 4.13 | *p* < 0.001 | 1>2>3 |
| CPRS-R:L hyperactive/impulse symptom severity T-score | 66.3 | 15.67 | 50.5 | 12.09 | 45.02 | 3.43 | *p* < 0.001 | 1>2>3 |
| CPRS-R:L total symptom severity T-score | 64.44 | 14.85 | 50.2 | 11.95 | 43.64 | 3.39 | *p* < 0.001 | 1>2>3 |
| Inattentive Symptom Count | 6.06 | 2.53 | 6.78 | 1.59 | - | - |  | - |
| Hyperactive/Impulsive Symptom Count | 4.72 | 2.55 | 3.67 | 2.54 | - | - | - | - |
| *Note:* *M:* mean, *SD*: standard deviation*,* 1 = ADHD, 2 = US, 3 = HC*,* Estimated IQ based on Wechsler Intelligence Scale for Children or Wechsler Adult Intelligence Scale-III Vocabulary and Block design. Multiple comparisons adjusted with Bonferroni. | | | | | | | | |

| *Table S2.*  Prevalence rates of substance use disorder and nicotine dependence in children with attention deficit hyperactivity disorder (ADHD), their unaffected siblings (US) and healthy controls (HC). | | | | | | | | | | | | | | | | | | | | | | | | |
| --- | --- | --- | --- | --- | --- | --- | --- | --- | --- | --- | --- | --- | --- | --- | --- | --- | --- | --- | --- | --- | --- | --- | --- | --- |
|  | Prevalence rates | | | | | | | |  | | Hazard ratios | | | | | | | | | | | |  |  |
|  | ADHD  *n* = 74 | |  | US  *n* = 68 | |  | HC  *n* = 47 | | |  | | | ADHD  vs.  HC | | |  | | US  vs.  ADHD | |  | US  vs.  HC | | | |
|  | *n* | % |  | *n* | % |  | *n* | % | |  | | HR | | 95% CI |  | | HR | | 95% CI |  | HR | 95% CI | | |
| Substance Use Disorder | 23 | 31.1 |  | 16 | 23.5 |  | 6 | 12.8 | |  | | 4.63* | | 1.43-14.99 |  | | .70 | | .35-1.38 |  | 3.23* | 1.07-9.78 | | |
| Nicotine Dependence | 17 | 23 |  | 13 | 19.1 |  | 2 | 4.3 | |  | | 6.34 | | 1.18-34.20 |  | | .87 | | .43-1.79 |  | 5.53 | .97-31.35 | | |
| *Note:* Hazard ratios (*HR*) were calculated using Cox proportional hazard regression. All comparisons were corrected for gender and follow-up interval in years. 95% *CI*: 95% confidence interval, *Significant at *p* < 0.05. | | | | | | | | | | | | | | | | | | | | | | | | |

| *Figure S1*: Cumulative life-time risk for any substance use disorder (SUD) and nicotine dependence in Attention-deficit hyperactivity disorder (ADHD) probands (*n* = 74), their unaffected siblings (*n* = 68) and healthy controls (*n* = 47) at baseline. All comparisons were corrected for gender and follow-up interval. |
| --- |

| *Table S3.*  Demographic and clinical characteristics of the Attention-deficit/hyperactivity disorder (ADHD) persistent, remittent, late-onset (LO), unaffected siblings (US) and healthy controls (HC) group. | | | | | | | | | | | |
| --- | --- | --- | --- | --- | --- | --- | --- | --- | --- | --- | --- |
|  | Persistent  *n* = 62 | | Remittent  *n* = 12 | | LO *n* = 18 | | US *n* = 50 | | HC *n* = 47 | |  |
|  | *M* | *SD* | *M* | SD | *M* | SD | *M* | SD | *M* | SD | Post-hoc |
| **Wave 1** |  |  |  |  |  |  |  |  |  |  |  |
| ***Demographic*** |  |  |  |  |  |  |  |  |  |  |  |
| Age | 11.64 | 2.83 | 10.94 | 1.88 | 9.25 | 3.88 | 11.43 | 2.66 | 11.49 | 3.08 | 1=2=3=4=5 |
| Gender, N male % | 47 | 75.8 | 9 | 75 | 10 | 55.6 | 22 | 44 | 21 | 44.7 | 1=2=3>4=5 |
| Estimated full-scale IQ | 98 | 12 | 98 | 12 | 95 | 18 | 102 | 10 | 110 | 10 | 1=2=3=4<5 |
| ***ADHD severity*** |  |  |  |  |  |  |  |  |  |  |  |
| CPRS-R:L inattentive symptom severity T-score | 71.42 | 9.14 | 70.67 | 9.2 | 55.11 | 8.69 | 49.26 | 8.8 | 45.22 | 3.91 | 1=2>3,4,5; 3>5; 3=4; 4=5 |
| CPRS-R:L hyperactive/impulse symptom severity T-score | 78.65 | 10.83 | 72.67 | 7.94 | 57.83 | 13.23 | 51.9 | 11.07 | 45.78 | 3.37 | 1=2>3=4>5 |
| Inattentive Symptom Count | 7.83 | .92 | 7.83 | 1.12 | - | - | - | - | - | - | - |
| Hyperactive/Impulsive Symptom Count | 8.1 | 1.43 | 7.5 | 1.57 | - | - | - | - | - | - | - |
| ***Anxiety and emotional lability severity*** |  |  |  |  |  |  |  |  |  |  |  |
| CPRS-R:L anxiety symptom severity T-score | 57.47 | 14.27 | 56.5 | 15.14 | 52.61 | 11.77 | 51.84 | 10.5 | 47.09 | 6.37 | 1=2>5; 1>4=5; 2=4; 3=1&2&4&5; |
| CPRS-R:L emotional lability symptom severity T-score | 65.03 | 13.95 | 61.83 | 15.47 | 52.11 | 10.35 | 49.9 | 11.25 | 45.5 | 6.35 | 1=2>3>5; 3=4; 4=5 |
| **Wave 4** |  | |  | |  | |  | |  | |  |
| ***Demographic*** |  |  |  |  |  |  |  |  |  |  |  |
| Follow-up period | 9.73 | .76 | 10.27 | .8 | 9.69 | .84 | 9.73 | .85 | 7.92 | .38 | 1=2=3=4>5 |
| Age | 21.07 | 2.92 | 21.17 | 3.35 | 19.23 | 4.05 | 21.43 | 3.22 | 19.42 | 3.33 | 4>5 |
| Estimated full-scale IQ | 97 | 19 | 105 | 20 | 101 | 19 | 107 | 15 | 114 | 13 | 1<4=5 |
| ***ADHD severity*** | | | | | | | | | | | |
| CPRS-R:L inattentive symptom severity T-score | 62.33 | 12.42 | 49.8 | 5.51 | 62.38 | 10.89 | 45.5 | 6.25 | 44.2 | 4.13 | 1=3>2=4=5 |
| CPRS-R:L hyperactive/impulse symptom severity T-score | 68.96 | 15.22 | 51.9 | 9.09 | 60.81 | 16.68 | 46.75 | 7.04 | 45.02 | 3.43 | 1=3>4=5; 1>2=4=5; 2=3 |
| Inattentive Symptom Count | 6.87 | 1.58 | 1 | .82 | 6.78 | 1.59 | .57 | .89 | .63 | 1.65 | 1=3>2=4=5 |
| Hyperactive/Impulsive Symptom Count | 5.32 | 2.18 | 1 | 1.05 | 3.67 | 2.54 | .64 | 1.03 | .35 | .69 | 1>3>2=4=5 |
|  |  |  |  |  |  |  |  |  |  |  |  |
| ***Anxiety and emotional lability severity*** |  |  |  |  |  |  |  |  |  |  |  |
| CPRS-R:L anxiety symptom severity T-score | 52.81 | 11.56 | 45.70 | 1.95 | 52.25 | 8.96 | 47.75 | 7.94 | 45.62 | 3.56 | 1>2=4=5; 3=1&2&4; 3>5 |
| CPRS-R:L emotional lability symptom severity T-score | 55.13 | 1.41 | 49.2 | 3.29 | 53.56 | 2.6 | 45.77 | 1.57 | 43.78 | 1.55 | 1=3>4=5; 2=1&3&4&5 |
| Note: *M:* mean, *SD:* standard deviation. 1 = Persistent, 2 = Remittent, 3 = LO, 4 = US, 5 = HC. Estimated IQ based on Wechsler Intelligence Scale for Children or Wechsler Adult Intelligence Scale-III Vocabulary and block design. Multiple comparisons adjusted with Bonferroni. | | | | | | | | | | | |
